# SRPS associated protein WDR60 regulates the multipolar-to-bipolar transition of migrating neurons during cortical development

**DOI:** 10.1038/s41419-020-03363-3

**Published:** 2021-01-12

**Authors:** Cui Li, Yu Zheng, Yufang Zheng, Zhiheng Xu

**Affiliations:** 1 State Key Laboratory of Molecular Developmental Biology, CAS Center for Excellence in Brain Science and Intelligence Technology, Institute of Genetics and Developmental Biology, Chinese Academy of Sciences, Beijing, China; 2 University of Chinese Academy of Sciences, Beijing, 100101 China; 3 Obstetrics & Gynecology Hospital, Institute of Reproduction & Development, Fudan University, Shanghai, 200011 China; 4 Institute of Developmental Biology & Molecular Medicine, State Key Laboratory of Genetic Engineering, School of Life Sciences, Fudan University, Shanghai, 200433 China; 5 Parkinson’s Disease Center, Beijing Institute for Brain Disorders, Beijing, 100053 China

**Keywords:** Cell migration, Embryogenesis

## Abstract

Mutations of WD40 repeat domain 60 (*WDR60*) have been identified in short-rib polydactyly syndromes (SRPS I–V), a group of lethal congenital disorders characterized by short ribs, polydactyly, and a range of extraskeletal phenotypes. However, the underlying mechanism is still unclear. Here, we report that WDR60 is essential for embryonic development and plays a critical role in the multipolar-bipolar transition and migration of newborn neurons during brain development. Mechanically, we found that WDR60 was located at the microtubule-organizing center to control microtubule organization and possibly, the trafficking of cellular components. Importantly, the migration defect caused by *Wdr60* knockdown could be rescued by the stable form of α-Tubulin, α-Tubulin^K40Q^ (an acetylation-mimicking mutant). These findings identified a non-cilia function of WDR60 and provided insight into its biological function, as well as the pathogenesis of WDR60 deficiency associated with SRPS.

## Introduction

The mammalian cerebral cortex is well ordered and hierarchical^1^. Depending on the morphology and function, the developing cerebral cortex cells are divided into three major layers, namely the cortical plate (CP), which is the neuronal region, the ventricular zone/sub ventricular zone (VZ/SVZ), and the intermediate zone (IZ). During embryonic development, proliferation and differentiation of neural progenitor cells (NPCs) mainly occur in the VZ/SVZ^1,2^. The first wave of post-mitotic neurons leave the VZ/SVZ region and move radially along the radial glial cells toward the surface to form preplate. Later born neurons pass through the previously generated cells to the outer region in an “inside-out” manner to form the CP layer. In the CP region, neurons begin to grow dendrites and axons to establish synaptic connections and form the cerebral cortex^3^.

Newborn neurons initially adopt a multipolar morphology with constant morphological rearrangement in the lower IZ. After undergoing the multipolar-to-bipolar transition in the upper IZ to form bipolar morphology with long leading processes, neurons start glial-guided locomotion and radially migrate toward the CP to reach their final positions in the neocortex^4–6^. Accumulating evidences have shown that impairment of neuronal migration leads to cortical malformations and severe neuropsychiatric disorders, such as lissencephaly, mental retardation, schizophrenia, and autism^7–9^.

Short-rib polydactyly syndrome (SRPS) is an autosomal recessive chondrodysplasia, which can be further divided into five subtypes. *WDR60* missense mutations have been detected in SRPS type III patients. SRPS manifest extremely shortened long bones, a small, narrow thorax, and frequent pre- and postaxial polydactyly. Other multisystem anomalies of SRPS, including cardiac malformations, anencephaly, and severe brain abnormalities were also observed^10,11^. However, the biological function of WDR60 during brain development and the underlying mechanism are still unclear.

WDR60 is a member of the WD40 repeat protein family. WD40 repeats facilitate the formation of heterotrimeric or multiprotein complexes and are involved in intracellular trafficking, cell cycle control, protein binding, cytoskeletal organization, and cargo recognition^12,13^. Several WD40 domain-containing proteins have been reported to play a crucial role in brain development. For example, mutations in WDR62 affect neurogenesis and cause microcephaly^14,15^ and deletions in LIS1 result in lissencephaly due to dysfunction of cytoplasmic dynein^16^. WDR60 was reported as bona fide dynein-2 intermediate chain that is required for dynein-2 function and is essential for retrograde ciliary trafficking and ciliogenesis^17^. Cilia are highly conversed microtubule-based organelles and critical for many signaling^18^.

In this study, we reported that knockdown of *Wdr60* disturbed multipolar-bipolar transition and neuronal migration in the developing neocortex. Furthermore, WDR60 interacted with α-Tubulin and was essential for microtubule growth. Our findings demonstrated that WDR60-mediated microtubule organization was required for neuronal migration.

## Materials and methods

### Animals

The pregnant rats and mice were bought from Beijing Vital River Laboratory Animal Technology Co., Ltd. The experimental procedures were performed according to protocols approved by the Institutional Animal Care and Use Committee at Institute of Genetics and Developmental Biology, Chinese Academy of Sciences.

*Wdr60* KO mouse was generated in FVB genetic background by piggyBac^19^. The primers for genotyping are:

$${\mathrm{PB}}:5^\prime - {\mathrm{CTGAGATGTCCTAAATGCACAGCG}} - 3^\prime ,$$

$${\mathrm{GL}}:5^\prime - {\mathrm{TCAGAGGTAGTCTTTGCCCACC}} - 3^\prime ,\;{\mathrm{and}}$$

$${\mathrm{GR}}:5^\prime - {\mathrm{CCCAAGCTGCTTGTTAGTTTGC}} - 3^\prime .$$

The primers used for quantitative real-time (qRT)-PCR to verify the *Wdr60* KO efficiency are

$${\mathrm{Wdr}}60 - {\mathrm{F}}:5^\prime - {\mathrm{TGAGTCGAGATATGCGTGGC}} - 3^\prime \;{\mathrm{and}}$$

$${\mathrm{Wdr}}60 - {\mathrm{R}}:5^\prime - {\mathrm{CTCTTCTCCCGATCTGCGTC}} - 3^\prime .$$

### Primary cortical neurons isolation and culture

Primary cortical neurons were isolated from embryonic 15.5 mice. Cortex was detached from E15.5 mice under microscope. After digested by 0.25% trypsin for 15 min, the reaction of digestion was stopped by DMEM containing 10% FBS. These cells were cultured with Neurobasal medium (Invitrogen) supplemented with 1% glutaMAX, 2% B27, and 1% PS.

### qRT-PCR

To detect the expression of genes, primary cortical neurons were cultured for 6 days in vitro (DIV 6). Total RNA was isolated from DIV 6 primary cultured neurons and E15.5 mice cortex. cDNA was prepared from 1 μg RNA by using GoScript Reverse Transcription System (Promega). For qRT-PCR, cDNA was mixed with SsoFast EvaGreen Supermix (Bio-Rad) and a couple of primers. The primers were as follows:

$${\mathrm{Wdr}}60 - {\mathrm{F}}:5^\prime - {\mathrm{TGAGTCGAGATATGCGTGGC}} - 3^\prime ,$$

$${\mathrm{Wdr}}60 - {\mathrm{R}}:5^\prime - {\mathrm{CTCTTCTCCCGATCTGCGTC}} - 3^\prime ;$$

$${\mathrm{Actin}} - {\mathrm{F}}:5^\prime - {\mathrm{AGGGAAATCGTGCGTGAC}} - 3^\prime ,$$

$${\mathrm{Actin}} - {\mathrm{R}}:5^\prime - {\mathrm{GATAGTGATGACCTGACCGT}} - 3^\prime .$$

PCR reactions were performed in 96-well plates with Bio-Rad CFX Connect Real-Time System (Bio-Rad).

### Plasmids

*Wdr60* small hairpin RNAs (shRNAs) were generated in pLL3.7 vector. The targeting regions of *Wdr60* were as follows:

$${\mathrm{sh}}60 - 1,\;5^\prime - {\mathrm{GTTGGCGAGTTATCTTTGAAA}} - 3^\prime ;$$

$${\mathrm{sh}}60 - 2,\;5^\prime - {\mathrm{CCGTGAGAAAGACAAGCTAAA}} - 3^\prime .$$

Scramble shRNA contained no homology to any known mammalian genes. FLAG-Wdr60 was generated by cloning mouse *Wdr60* cDNA sequence with a FLAG tag in the N terminal into the pCMS-EGFP vector. Its sh60-1 resistant synonymous mutation FLAG-Wdr60^R^ was generated into both pCMS-EGFP and pCAGIG-EGFP vectors. The mutated sequence is 5′-GTTGGCGAATTGTCATTGAAA-3′. The plasmid Tubulin^K40Q^, mimicking acetylated α-Tubulin was kindly provided by Dr. Lan Bao^20^.

### In utero electroporation

IUE was performed as described previously^14,21^. Briefly, plasmid DNA plus fast green was injected into the lateral ventricle of the embryonic brain with a glass micropipette. 5 × 50 ms, 42 V square pulses for rats and 35 V for mice were delivered for electroporation. For rescue analysis, sh60-1 was mixed with rescue construct and injected into mouse brains. Sh60-1 and pCAGIG-EGFP-Wdr60^R^ were mixed in a 1:2 ratio, and sh60-1 together with Tubulin^K40Q^ were mixed in a 1:1 ratio.

### Time Lapse

Embryonic mouse brains were electroporated at E14.5 and sacrificed 2 days later. After checking green fluorescence under microscope, the embryonic brain was sectioned into 300 μm in artificial cerebrospinal fluid. The cortical slices were transfered onto Millicell inserts (Millipore) in Neurobasal medium (Invitrogen) containing 2% B-27 supplement, 2 mM L-glutamine, and 1% penicillin/streptomycin. The inserts were placed into a glass-bottomed dish for microscope observation.

### Immunostaining

For immunostaining, the brains were fixed in 4% paraformaldehyde (PFA) overnight at 4 °C, then dehydration in 30% sucrose, and frozen in tissue freezing medium. Brains were sectioned into 40 µm and then used for immunostaining as described previously^14,21^.

The antibodies used for immunostaining were SOX2 (Abcam, ab97959, 1:1000), β-III Tubulin (TUJ1) (Abcam, ab7751, 1:1000), γ-Tubulin (Abcam, ab11316, 1:1000), ODF2 (Abcam, ab43840, 1:1000), α-Tubulin (CST, 2144 s, 1:1000), Ace-α-Tubulin (Abcam, ab24610, 1:1000), WDR60 (Sigma, HPA020607, 1:200), Ctip2 (Abcam, ab18465, 1:1000), Caspase3 (Abcam, ab13847, 1:1000). Nuclei were stained with DAPI (Invitrogen). Images were captured by LSM 700 (Carl Zeiss) confocal microscope, and analyzed with Imaris and ImageJ.

### Microtubule depolymerization and regrowth

Cells were treated with 2 μg/mL nocodazole for 1 h. After removing nocodazole, cells were washed with PBS, and then replaced with fresh medium for 0 min, 10 min, 30 min and 1 h respectively. Cells were fixed with 4% PFA for 15 min at room temperature, and followed by immunostaining.

### MEF isolation and culture

MEF was isolated from embryonic mice at E10.5. The head and organs of fetal mice were removed under microscope and the left tissue was digested by 0.25% trypsin for 10 min. Culture media is DMEM + 10%FBS + 1%PS.

### Western blotting and immunoprecipitation

The procedure of western blotting and immunoprecipitation was performed as previously described^22^. The antibody used for immunoblotting were as followed GAPDH (MBL, M171-3, 1:3000), FLAG (MBL, M185-3L, 1:3000), α-Tubulin (CST, 2144 s, 1:5000), Ace-α-Tubulin (Abcam, ab24610, 1:3000), WDR60 (Sigma, HPA020607, 1:1000).

### Statistical analysis

Image quantifications were performed by researchers blinded to the group allocation. All the rats and mice used were randomly picked from the nest. Brain sections from similar positions were randomly chosen for immunostaining. All data were analyzed using Prism software (GraphPad) or Excel.

## Results

### WDR60 is expressed in immature neuron and regulates neuronal migration during brain development

**Fig. 1:**
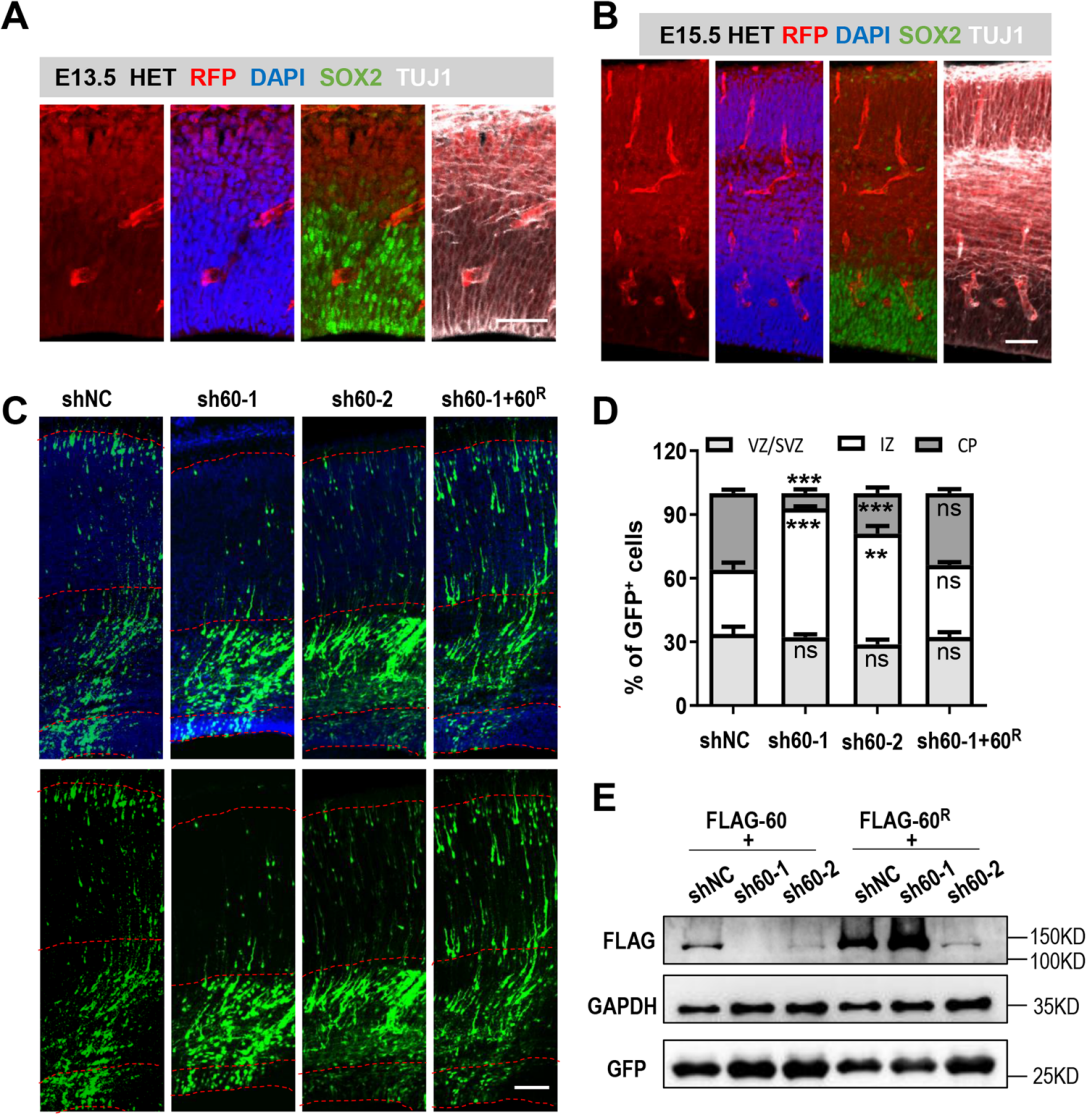
WDR60 is expressed in the developing neocortex and essential for neural migration. **a**, **b** Images of cortex slices from E13.5 (**a**) and E15.5 (**b**) *Wdr60* HET mice immunostained for SOX2 (red), TUJ1 (white) and DAPI (blue). Scale bar, 50 µm. **c** Images of rat cortical sections electroporated in utero with the indicated constructs at E16.5 and examined at E20.5. Scale bar, 100 µm. **d** Quantification of the distribution of EGFP^+^ cells in E20.5 cortical sections. n: 6 slices from 3 mice. **e** Indicated constructs were transfected into HEK293T cells. 48 h later, cell lysates were collected for verifying knockdown efficiency of shRNAs and the resistant of *Wdr60*^*R*^ to sh60-1. All data are means ± SEM, *t*-test. **p < 0.01, ***p < 0.001.

To elucidate the role of WDR60 during brain development, we obtained a mouse line in which a DNA fragment containing RFP sequence was inserted in the intron between the 3rd and 4th exons of *Wdr60* by PB transposon (Supplementary Fig. S1A)^19^. Knockout of *Wdr60* was confirmed at mRNA level (Supplementary Fig. S1B), and homozygous mutants were embryonic lethal (manuscript in preparation). We then inspected the expression of WDR60 in the developing neocortex. Because WDR60 antibody was not available for mouse tissue, we took advantage of the heterozygote mice that express RFP in WDR60 expressing cells in vivo. We found that at embryonic day (E)13.5 and E15.5, WDR60 was expressed at high levels in the IZ and CP. RFP signal co-localized with TUJ1^+^ immature neurons, but not with SOX2^+^ NPCs (Fig. 1a, b). Immunostaining with RFP antibody showed similar results (Supplementary Fig. S1C). We also confirmed by RT-PCR that WDR60 was expressed in both cortex from E15.5 and cultured neurons (Supplementary Fig. S1D).

Because WDR60 is expressed in the IZ and CP where the immature neurons are located^1^, we explored whether WDR60 is required for the migration of neuron. We generated two shRNA expressing vectors (sh60-1 and sh60-2), targeting different regions of rat/mouse *Wdr60* to efficiently downregulate its expression. These shRNA vectors could also express EGFP under an independent promoter for the observation of transfected cells. We electroporated the vectors into the rat embryonic cortex via *in utero electroporation* (IUE) at E16.5 and inspected the distribution of EGFP^+^ cells at E20.5, during which extensive migration occurs^1^. Both sh60-1 and sh60-2 led to a significant increase of EGFP^+^ cells in the IZ, but a decrease in the CP (Fig. 1c, d). The severity of migration defects was correlated with the knockdown efficiency of shRNA vectors (Fig. 1e). To exclude the possibility of off-target effects and to recover the expression of *Wdr60*, a construct, *Wdr60*^*R*^, which is resistant to sh60-1, was developed by synonymous mutation of three base pairs targeted by sh60-1. The migration defect was rescued by *Wdr60*^*R*^ (Fig. 1c, d).

### Loss of WDR60 perturbs the multipolar-to-bipolar transition of migrating neurons

**Fig. 2:**
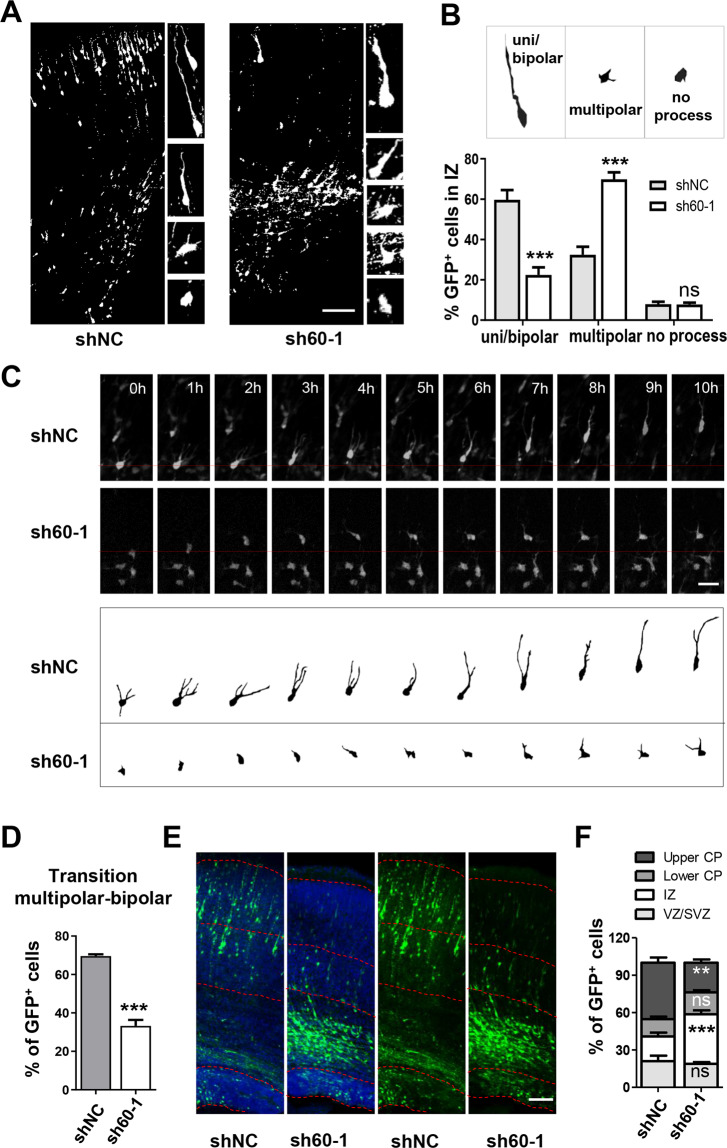
*Wdr60* knockdown impairs the multipolar-to-bipolar transition of migrating neurons. **a**, **b** Images of rat cortical sections electroporated in utero at E16.5 and examined at E20.5 (**a**). Scale bar, 100 µm. **b** Quantification of EGFP^+^ cells exhibiting different morphology in the IZ. Upper panel: the cell morphology was delineated based on EGFP fluorescence. *n*: 6 slices from 3 mice. **c** Representative images of live imaging. E14.5 mouse embryos were electroporated with scramble or sh60-1. Cortical slices were prepared at E16.5, and the migration of EGFP^+^ neurons were observed for 10 h. Lower panel: neurons were delineated based on the cells labeled by red line at 0 h in the upper panel. Scale bars: 30 μm. **d** Quantification of EGFP^+^ cells that completed multipolar-to-bipolar morphology transition during the recording period. shNC *n* = 4, sh60-1 *n* = 3. **e**, **f** Images of rat cortical sections electroporated in utero at E16.5 and examined 6 days later (E22.5 or P0) (**e**). Scale bar: 100 μm. **f** Quantification of the distribution of EGFP^+^ cells. n: 6 slices from 3 mice. All data are means ± SEM, *t*-test. **p < 0.01, ***p < 0.001.

To decipher the mechanisms underlying the migration defect, we inspected the newborn neurons in more detail. During neocortex development, post mitotic neurons first undergo slow migration in a multipolar morphology and later transform to bipolar shape with constant morphological rearrangement in the IZ^4–6^. Four days after electroporation at E16.5 (rat), about 60% cells in IZ had acquired the bipolar shape with the leading processes extending toward the pia in the scramble group (Fig. 2a, b). In contrast, knockdown of *Wdr60* increased the proportion of multipolar neurons substantially. *Wdr60* knockdown neurons had irregular morphology with poorly developed shorter processes (Fig. 2a, b).

We then visualized the morphology defect and migration process within the IZ by time-lapse imaging. Considering the limitation in the view of brain slice, we electroporated shRNA at E14.5 and examined at E17.5 in mouse. We confirmed that the migration defect was consistent and the significant reduction of Ctip2^+^ cells caused by *Wdr60* knockdown (Supplementary Fig. S2A, B). We then performed electroporation in mice at E14.5, and observed the living cortical slices 2 days later. Live images showed the clear transition from multipolar to bipolar and migration in controls. *Wdr60* knockdown neurons failed to form an obvious leading process and were arrested at the multipolar stage, leading to slower migration (Fig. 2c, d).

To exclude the impact of transfection efficiency or cell death caused by shRNAs, we counted the number of EGFP^+^ cells and stained with Caspase3 antibody 24 h after electroporation and found the results were similar (Supplementary Fig. S2C, D). We also electroporated shRNAs in the E16.5 rat cortex and observed the migration 6 days later (E22.5 or P0). Most control EGFP^+^ cells migrated to the CP, with approximately 50% located in the upper CP; however, most *Wdr60* knockdown neurons were still in the IZ, and only a few cells reached the upper CP (Fig. 2e, f). Together, these results showed that knockdown of *Wdr60* impaired the multipolar-to-bipolar transition of migrating neurons, which was correlated with the migration defects.

### WDR60 is located in the pericentrosome and controls microtubule organization

**Fig. 3:**
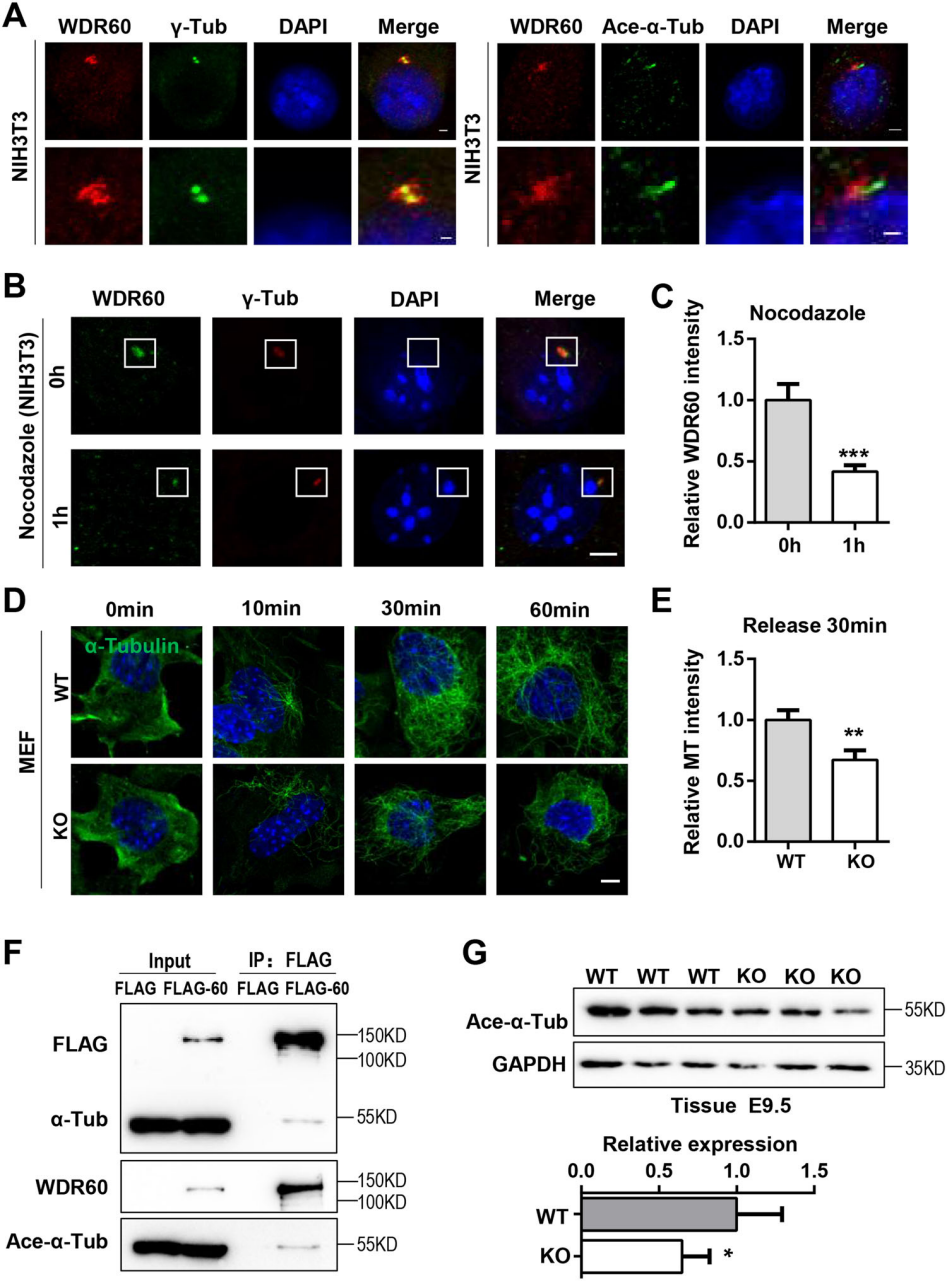
Loss of WDR60 affects microtubule organization. **a** NIH3T3 cells were immunostained for WDR60 (red) and γ-Tubulin (green) or Ace-α-Tubulin (green). Scale bars: 2 μm (upper), 1 μm (lower). **b**, **c** Imagines of NIH3T3 cells were treated or untreated with nocodazole (2 μg/ml) for 1 h and co-stained for WDR60 (green) and γ-Tubulin (red). Scale bars: 5 μm. **c** Quantification of WDR60 intensity. 0 h *n* = 43, 1 h *n* = 48. **d**, **e** WT and *Wdr60* KO MEF cells were treated with nocodazole for 1 h before release. Cells were fixed at different time points and stained for α-Tubulin (green). Scale bar: 5 μm. **e** Quantification of α-Tubulin intensity after released for 30 min. *n* = 54. **f** FLAG-Wdr60 was expressed in HEK293T cells and immunoprecipitated with FLAG beads and blotted for FLAG, α-Tub, Acetylated-α-Tub and WDR60. **g** Mouse tissues from WT and *Wdr60* KO at E9.5 were immunoblotted for Ace-α-Tubulin and GAPDH. Lower panel: quantification of Ace-α-Tubulin expression. All data are means ± SEM, *t*-test. *p < 0.05, **p < 0.01, ***p < 0.001.

To explore how WDR60 regulates migration, we inspected the subcellular location of WDR60 in NIH3T3 cells. Immunostaining of WDR60 indicated high expression in the pericentrosome, which is the microtubule organizing center (MTOC) labeled by γ-Tubulin (Fig. 3a). Moreover, WDR60 was also located at the basal body of cilia, consistent with its function in ciliogenesis^10^. The expression levels of WDR60 were reduced significantly after depolymerization of the microtubule by treatment with nocodazole, suggesting that the location of WDR60 was dependent on microtubule (Fig. 3b, c).

Cultured MEF cells from WT and *Wdr60* KO mice were also treated with nocodazole for 1 h to depolymerize microtubules. After washing out nocodazole, we visualized microtubule regrowth at different time points. The progress of regrowth was significantly delayed in knockout cells, indicating that microtubule reorganization was affected (Fig. 3d, e), although the number and structure of centrosomes and mother centriole were largely normal (Supplementary Fig. S3). In addition, coimmunoprecipitation indicated that WDR60 interacted with both total α-Tubulin and Ace-α-Tubulin (Fig. 3f). Furthermore, the level of Ace-α-Tubulin in the knockout tissue was significantly reduced. These results indicated that WDR60 bound to microtubules and may regulate microtubule stability (Fig. 3g).

### Expression of Tubulin^K40Q^ can largely recue the migration defects caused by WDR60 deficiency

**Fig. 4:**
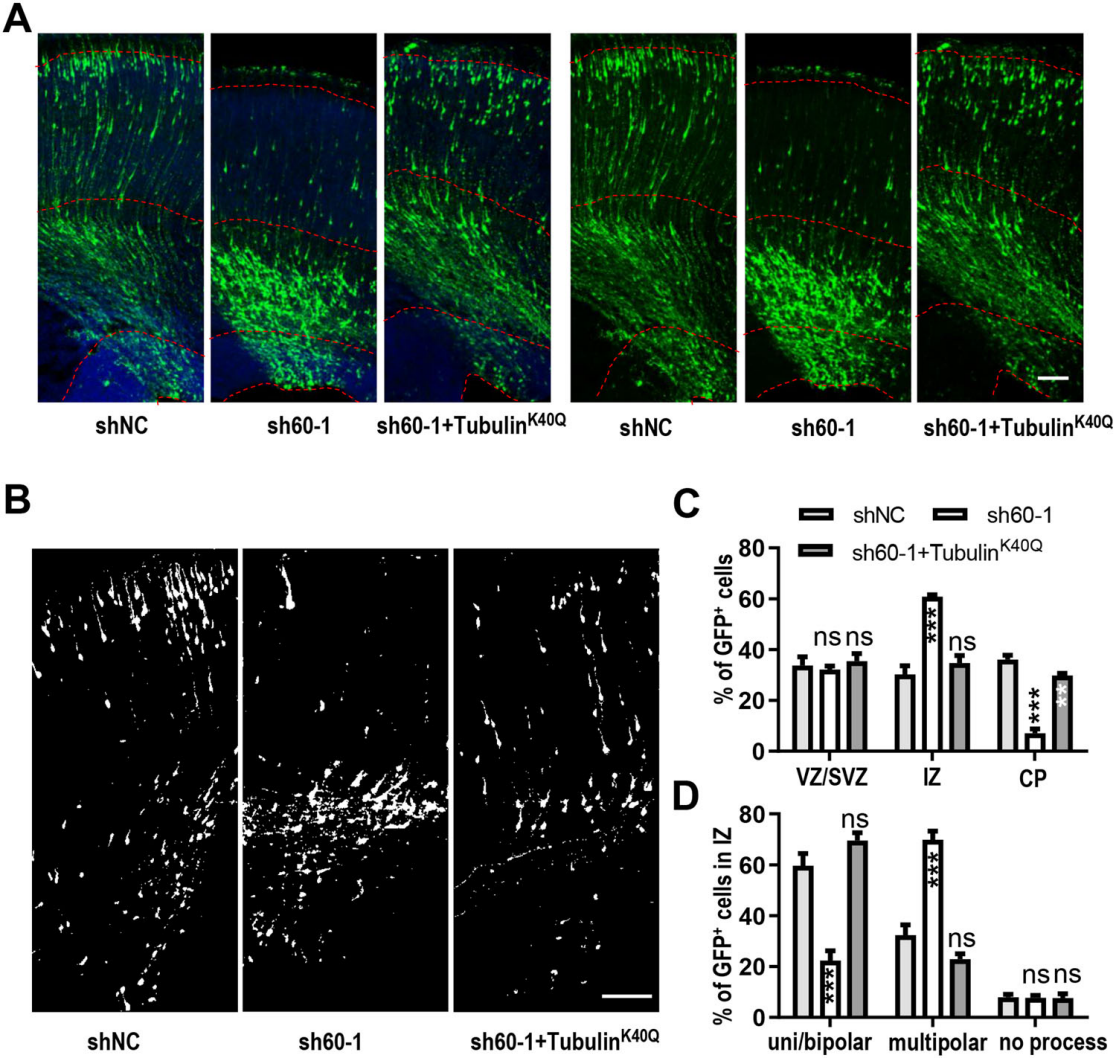
Mimicked Acetylated-α-Tubulin can rescue the migration defects caused by WDR60 deficiency. **a**, **b** Images of rat cortical sections electroporated in utero with the indicated constructs at E16.5 and examined at E20.5. **b** Images of transfected cells. Scale bars, 100 μm. **c** Quantification of the distribution of EGFP^+^ cells. *n*: 6 slices from 3 mice. **d** Quantification of EGFP^+^ cells exhibiting different morphology in the IZ. *n*: 6 slices from 3 mice. All data are means ± SEM, *t*-test. **p < 0.01, ***p < 0.001.

To confirm whether microtubules are involved in the migratory and morphological defects caused by knockdown of *Wdr60*, we co-expressed sh60-1 with Tubulin^K40Q^ at E16.5 (rat). Tubulin^K40Q^ is an acetylation-mimicking mutant, and has no effect on normal neural migration by itself^20,23^. At E20.5, we quantified the distribution of EGFP^+^ cells. Arrest of cells in the IZ was significantly rescued and cells migrated to the CP normally (Fig. 4a). Importantly, the multipolar morphology was also rescued (Fig. 4b–d). This results indicated that microtubules played an important role in the multipolar-to-bipolar transition and migration of newborn neurons.

## Discussion

In this study, we investigated the biological function of WDR60 and found that it was associated with microtubule organization. In addition, WDR60 was essential for the multipolar-bipolar transition and migration of newborn neurons during neocortical development.

Microtubule-associated proteins and tubulin modifications combine to regulate microtubule stability. Microtubule-associated proteins, LIS1 and doublecortin (DCX), have been shown to stabilize microtubules and regulate neuronal migration. Depletion of LIS1 or DCX causes the accumulation of multipolar neurons and migratory defects in cortical neurons^24–27^. In this study, we showed that WDR60 located at microtubule organizing center and interacted with tubulin. Its expression was also found to be dependent on the stability of microtubule. On the other hand, WDR60 deficiency led to the delay of microtubule reorganization, implicating its role in neuronal morphology transition. In support of this finding, expression of acetylation α-tubulin mimicking mutant rescued the multipolar-bipolar transition and migration defect of newborn neuron caused by WDR60 deficiency. Cilia were also microtubule-based organelle, originating from the parent centriole (basal body)^18^. WDR60 has been shown to be essential for retrograde ciliary trafficking and ciliogenesis^17^. Therefore, WDR60 deficiency may not only affect microtubule in cilia as a dynein component, but also disturb the dynein motor function of cytoplasmic microtubules.

WDR60 is localized in basal body of cilia and cilia play a role in cell cycle as a check point, which is important for normal cell cycle exit^28–30^. Interestingly in our study, *Wdr60* knockdown cells mainly accumulated in the IZ and failed to migrate to the CP. We used time-lapse imaging to visualize the migration process in the IZ and confirmed defects in morphology transition and migration in WDR60 deficient cells. Taken together, the results suggested that WDR60 played a specific role in the IZ and CP, which may be unrelated to its potential role in NPC proliferation and differentiation. Future studies are need to investigate whether *Wdr60* ablation leads to ciliary deficiency and abnormal proliferation and/or differentiation of NPCs.

In the current study, we found that WDR60 played an essential role in the multipolar-bipolar transition and migration of newborn neurons in the neocortex during development. Our findings also demonstrated a non-cilia function of WDR60, which involved the control of microtubule organization and possibly the trafficking of cellular components within migrating neuron during brain development.

## Supplementary information

Figure S1

Figure S2

Figure S3

Supplementary Figure Legends
