# Supplementary figures and images for: SRPS associated protein WDR60 regulates the multipolar-to-bipolar transition of migrating neurons during cortical development
Source: Cell Death Dis. 2021 Jan 12;12(1):75. doi: 10.1038/s41419-020-03363-3 (PMC7804399; doi:10.1038/s41419-020-03363-3)

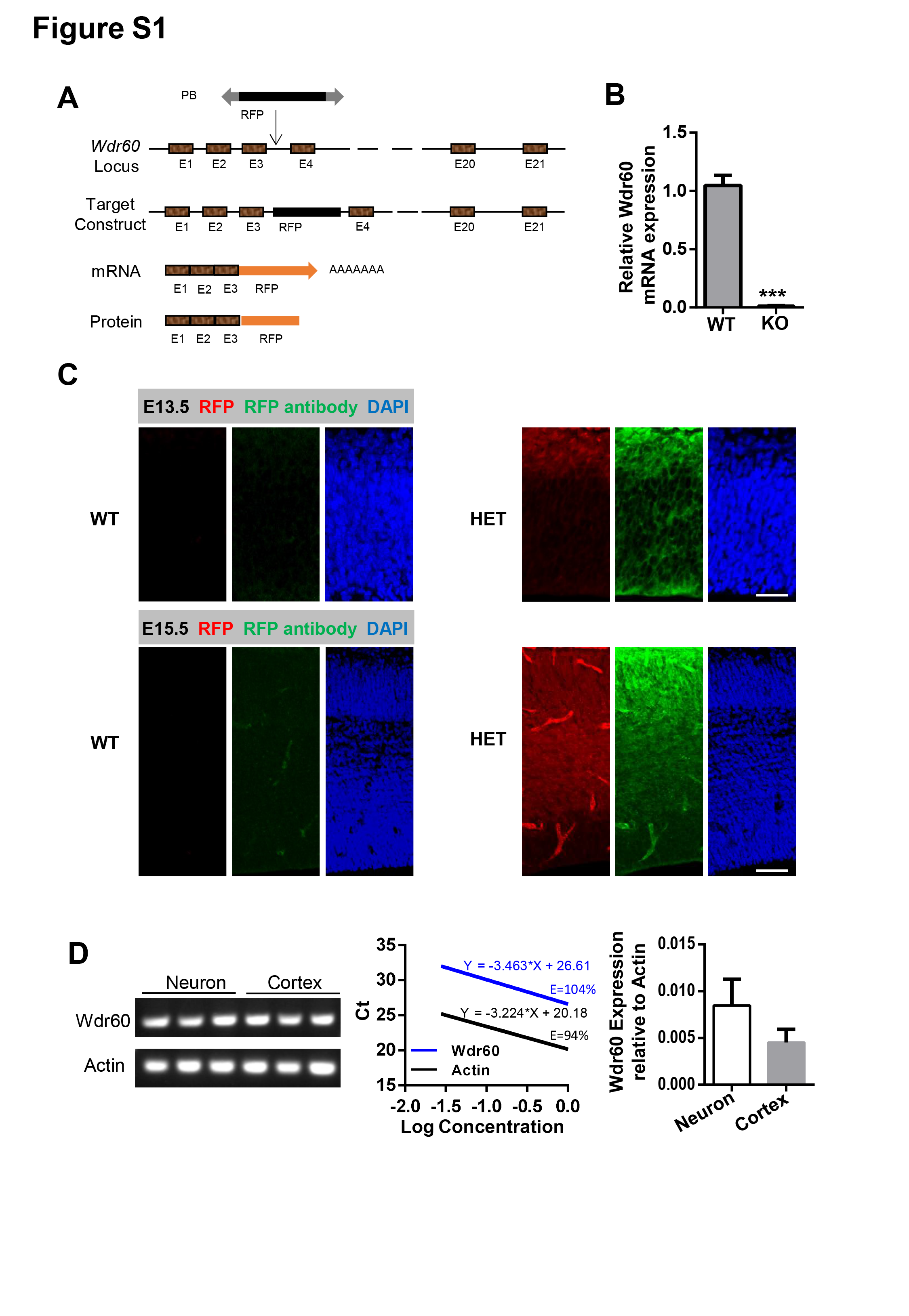

Supplement: Supplementary file 1 — Figure S1 [file 41419_2020_3363_MOESM1_ESM.tif]

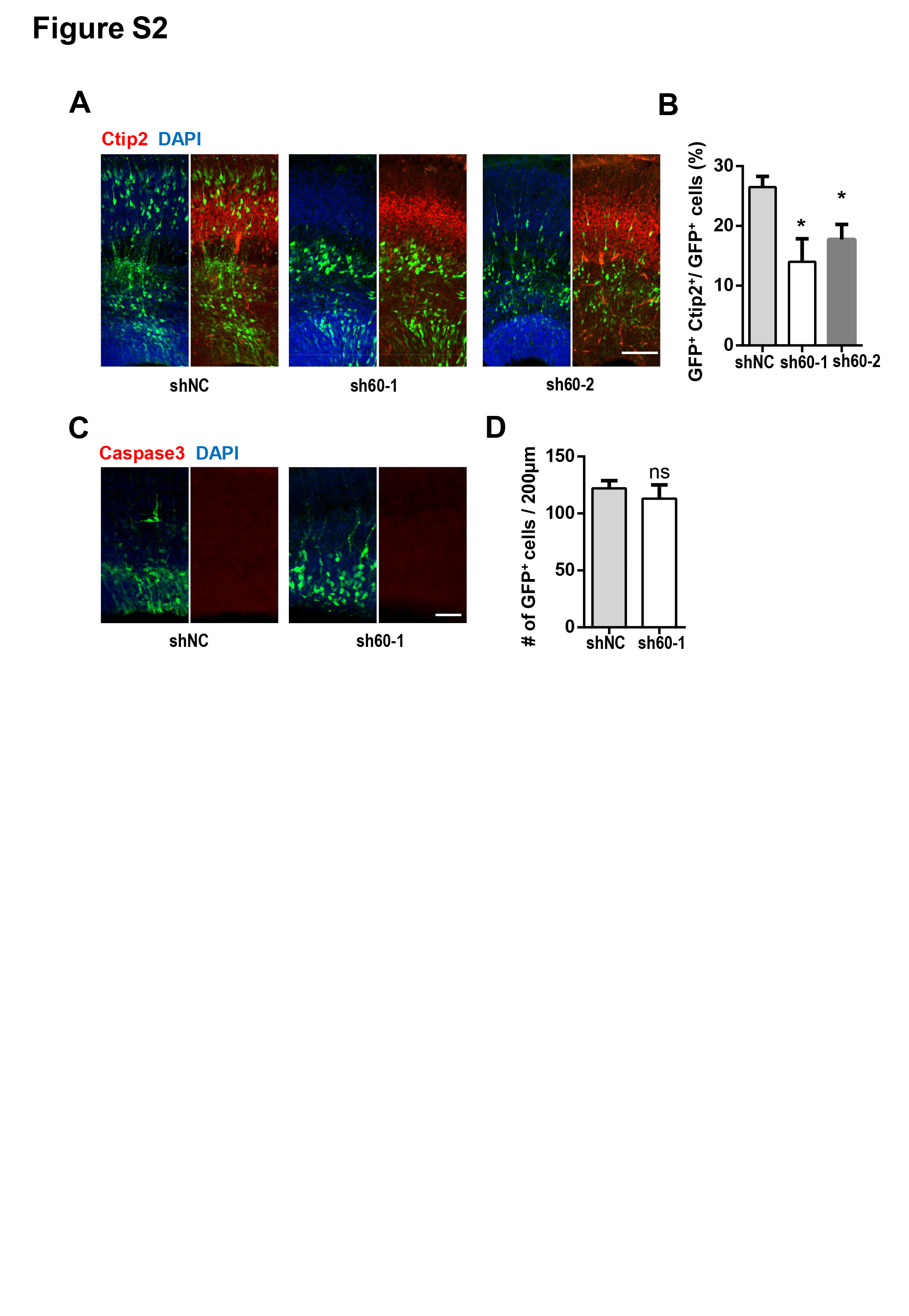

Supplement: Supplementary file 2 — Figure S2 [file 41419_2020_3363_MOESM2_ESM.tif]

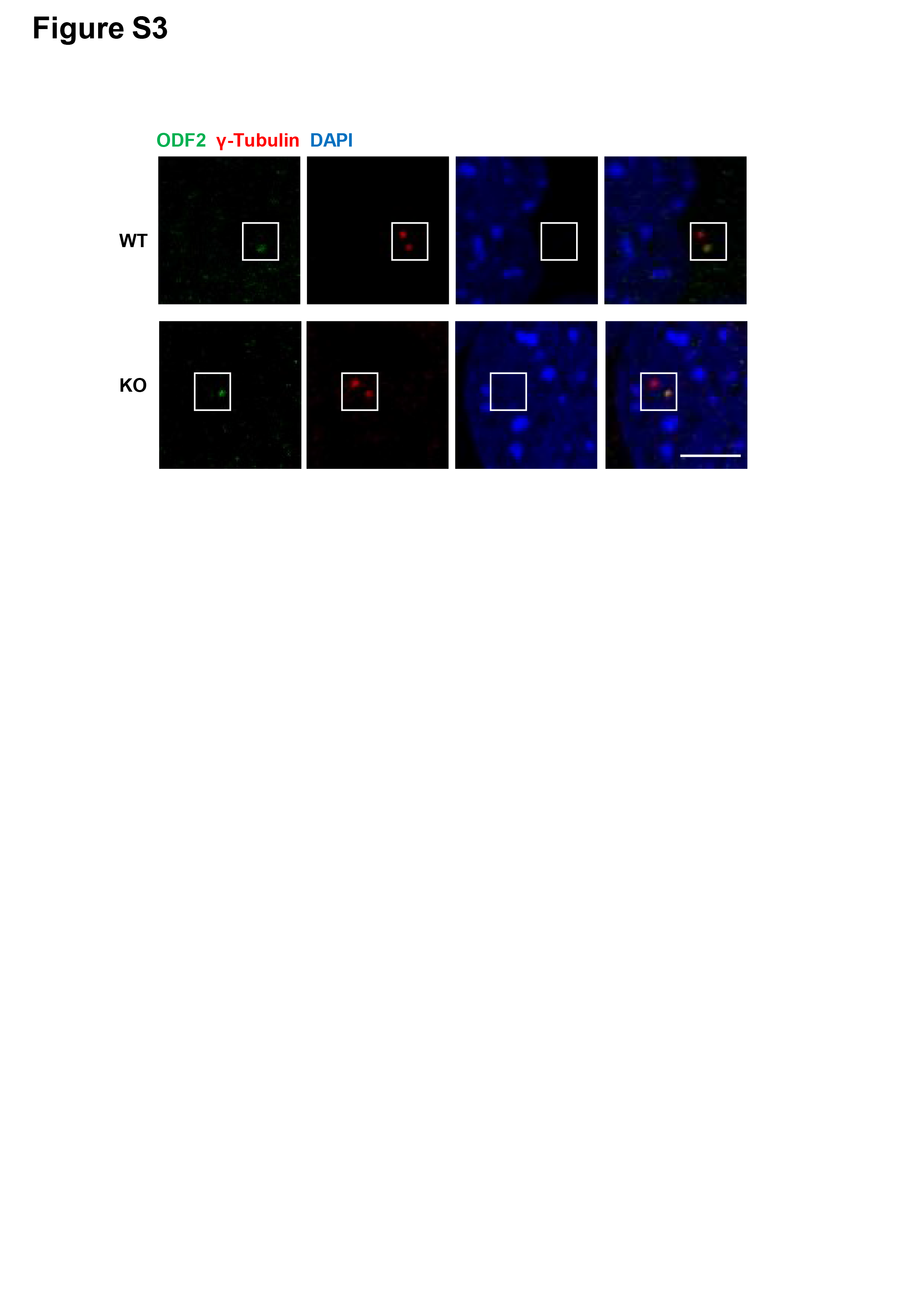

Supplement: Supplementary file 3 — Figure S3 [file 41419_2020_3363_MOESM3_ESM.tif]
