# Supplementary material for: SRPS associated protein WDR60 regulates the multipolar-to-bipolar transition of migrating neurons during cortical development
Source: Cell Death Dis. 2021 Jan 12;12(1):75. doi: 10.1038/s41419-020-03363-3 (PMC7804399; doi:10.1038/s41419-020-03363-3)
Supplement: Supplementary file 4 — Supplementary Figure Legends [file 41419_2020_3363_MOESM4_ESM.docx]

**Figure S1. WDR60 is expressed in immature neuron.**

1. Strategy for the generation of *Wdr60* KO mice. **(B)** *Wdr60* KO efficiency was examined by mRNA expression. n=3. **(C)** Images of cortex slices from E13.5 and E15.5 WT or *Wdr60* HET mice immunostained with RFP antibody (green). Scale bars: 30 μm (upper), 50 μm (lower). **(D)** *Wdr60* mRNA expression was confirmed in primary cultured neurons and cortex from E15.5 mice brain by qRT-PCR. n=3. All data are means ± SEM, t test. ***p < 0.001.

**Figure S2. *Wdr60* knockdown affects neural migration.**

**(A)** Images of mouse cortical sections stained with Ctip2. Indicated constructs were electroporated in utero at E14.5 and examined at E17.5. Scale bar, 100 µm. **(B)** Quantification of the ratio of EGFP and Ctip2 double positive cells to total EGFP^+^ cells. n: 6 slices from 3 mice. **(C, D)** Images of mouse cortical sections stained with Caspase3. Indicated constructs were electroporated in utero at E14.5 and examined at E15.5. Scale bar, 50 µm. (D) Quantification of the EGFP^+^ cells number in 200 µm width. n: 6 slices from 3 mice. All data are means ± SEM, t test. *p < 0.05.

**Figure S3. Normal Centrosome in *Wdr60* KO MEF.**

MEFs isolated from E10.5 WT and *Wdr60* KO mice were stained for γ-Tubulin (red) and ODF2 (green). Scale bar, 5 μm.
